# Technical and biological complications of implant-supported fixed complete dentures: a retrospective cohort study with up to 17 years of follow-up

**DOI:** 10.1186/s40729-026-00689-z

**Published:** 2026-05-13

**Authors:** Djan Pelser, Markus Schepers, Samir Abou-Ayash, Monika Bjelopavlovic, Mark K. Bremer, Leonie Grander, Stefan Wentaschek

**Affiliations:** 1 Department of Prosthetic Dentistry and Material Science, University Medical Center of the Johannes Gutenberg University Mainz, Augustusplatz 2, 55131 Mainz, Germany; 2 Institute for Medical Biostatistics, Epidemiology and Informatics, University Medical Centre, Johannes Gutenberg University Mainz, Rhabanusstraße 3, Bonifazius-Turm A, 55118 Mainz, Germany; 3 Department of Reconstructive Dentistry and Gerodontology, School of Dental Medicine, University of Bern, Freiburgstrasse 7, Bern, 3007 Switzerland

**Keywords:** Dental implants, Edentulous jaw, Dental prosthesis, Implant-supported, Treatment outcome, Peri-implantitis, Postoperative complications, Dental restoration failure, Full-arch, Prosthesis survival, Veneer fracture

## Abstract

**Purpose:**

To retrospectively evaluate biological and technical complication rates of implant-supported fixed complete dentures (IFCDs) in edentulous jaws and to identify factors associated with complications over long-term follow-up.

**Methods:**

Between 2003 and 2023, 91 IFCDs supported by 498 implants were placed in 72 patients. Mean observation period was 6.8 years (0.5–17). Biological and technical complications were compared between materials. Time until first complication was estimated using Kaplan–Meier analysis and risk factors for recurrent complications were assessed through multivariable Andersen–Gill Cox regression.

**Results:**

Seven IFCDs failed, corresponding to a cumulative overall IFCD survival of 92.3%. Twenty-one of 498 implants (4.2%) were explanted. Overall prosthesis survival of resin veneered (RV) and ceramic veneered (CV) IFCDs did not differ (*p* = 0.85), whereas veneer fracture–free survival was significantly higher for CV IFCDs (*p* = 0.0094). In total, 169 complications were recorded, including recurrent events, whereas 49.5% of prostheses remained complication-free. Technical complications predominated, with veneer fractures representing the most frequent event. Biological complications such as peri-implantitis and implant loss occurred less frequently. Compared to base metal alloy–ceramic IFCDs, titanium–resin IFCDs exhibited a significantly higher overall complication risk (HR 4.25, *p* = 0.0015), particularly for veneer fractures (HR 7.11, *p* = 0.0029).

**Conclusions:**

Within the limitations of this long-term retrospective cohort study, IFCDs demonstrated high prosthesis and implant survival rates, but a considerable number of predominantly technical complications. The choice of framework and veneering material appears to influence long-term complication risk and should be carefully considered during treatment planning.

**Graphical abstract:**

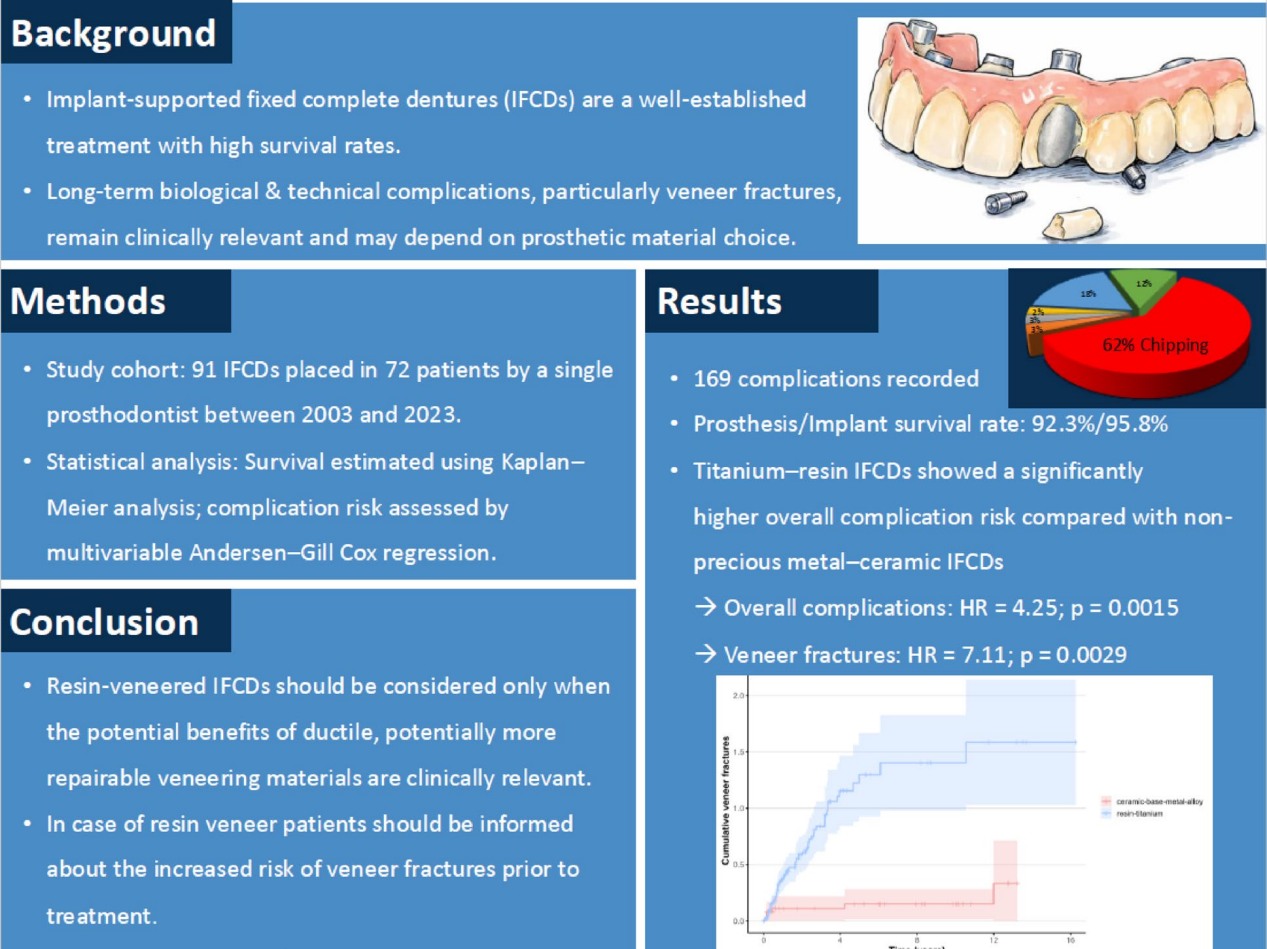

## Purpose

Edentulism continues to represent a major global health concern. According to estimates by the World Health Organization (WHO), approximately 350 million people are affected, with prevalence increasing particularly among aging populations [1, 2]. Epidemiological data indicate that both the number of edentulous patients and the application of implants have steadily increased over the past decades [3]. This development has been accompanied by an increase in implant-associated complications [4]. As the number of edentulous individuals continues to grow, the demand for functional and aesthetic implant-based rehabilitation will steadily increase [5]. Implant-supported fixed complete dentures (IFCDs) are considered an established treatment option, as they lead to significant improvements in chewing function and quality of life, addressing the functional and psychosocial consequences of edentulism, including impairments in mastication, phonetics, and swallowing as well as reduced oral sensibility and diminished self-esteem. Restoring these functions through IFCDs plays a crucial role in regaining well-being and social participation [6, 7]. Current evidence demonstrates that implant therapy remains a predictable and reliable treatment option even in older adults, who have the highest likelihood of edentulism [8]. Overall, dental implants represent a dependable and long-term successful therapeutic option for older individuals [8, 9]. The current ITI Consensus Report emphasizes that completely edentulous patients benefit substantially from both fixed IFCDs and removable implant overdentures (IODs) [10]. Both treatment modalities enhance quality of life, oral function, and tissue preservation compared to removable complete dentures. Different clinical, patient-related, and economic factors need to be considered to make the ideal choice between these two options. Treatment selection should consider prosthetic space requirements, maintenance demands, and patient-related factors such as manual dexterity and the ability to maintain adequate plaque control, as fixed solutions can make daily oral hygiene difficult for patients with limited manual dexterity [10]. However, evidence indicates that IFCDs generally yield greater improvements and are associated with higher levels of patient satisfaction, especially when it comes to stability and comfort [6]. Therefore, whenever feasible based on the aforementioned criteria, IFCDs are recommended, as they provide superior stability, retention, and comfort compared to IODs [10]. Despite the high survival rates reported in numerous studies, IFCDs are not without challenges. Their long-term prognosis is influenced not only by biological but also by technical issues, maintenance and material choice [11]. Metal–ceramic and titanium-based resin-veneered full-arch constructions continue to be considered materials of choice because of their well-documented long-term outcomes and reliable survival rates [12]. Nevertheless, a considerable number of technical complications with both material combinations have been demonstrated [13].

The primary aim of this retrospective clinical study was therefore to investigate the long-term complication rates and survival of IFCDs in order to assess the predictability of long-term stability and prosthetic prognosis. For this purpose, we tested the null hypothesis that resin-veneered (RV) IFCDs would exhibit complication rates comparable to those of ceramic-veneered (CV) IFCDs, and that overall IFCD survival, defined as the prosthesis remaining in situ and functional, would not differ significantly between the two groups. Secondary analyses focused on potential influencing factors associated with the occurrence of biological and technical complications, including jaw region (maxilla vs. mandible), opposing dentition, FP-Type, presence of cantilever, implant system, implant connection (butt-joint vs. conical connection), prosthetic level (abutment vs. implant level), number of implants per IFCD, complication location (anterior vs. posterior), framework and veneering materials—categorized into the two largest material groups of the present cohort (metal–ceramic and titanium–resin)—as well as patient-related factors such as sex and head and neck tumor disease.

## Methods

### Study design and population

The present retrospective single-center cohort study was conducted at the Department of Prosthodontics and Material Science, University Medical Center Mainz, and approved by the Ethics Committee of the State Medical Association of Rhineland-Palatinate (application number: 2023-17087-retrospective, approval date: July 13, 2023). All patient data were obtained from electronic patient records, and subsequently pseudonymized, preventing any identification of individual persons. Therefore, separate patient consent was not required. Data analysis was conducted exclusively for scientific purposes in accordance with applicable data protection regulations and was performed and reported in compliance with the STROBE guidelines.

The inclusion period covered 20 years (2003–2023). Prosthetic treatment and follow-up for all included patients were performed by the same experienced senior clinician from the Department of Prosthodontics. Surgical implant placement was carried out by specialists at the Department of Oral and Maxillofacial Surgery—Plastic Surgery, University Medical Center Mainz. All patients who received IFCDs that met the predefined inclusion criteria were included. These criteria included immediately and conventionally loaded implants, as well as patients with and without bone augmentation. Implant surgery was standardized to the extent that all patients were treated under local anesthesia, applying a type of CAIS [14]. The prosthetic protocol included, in all cases, an open-tray impression using polyether impression materials, followed by a try-in after jaw relation determination. Exclusively screw-retained restorations were delivered. All resin-veneered titanium frameworks in this study were milled and all IFCDs were either screwed directly onto the implant or onto a multi-unit abutment. No other abutment/connection types were used.

### Inclusion criteria

The following inclusion criteria were applied:

- Age ≥ 18 years at time of implant treatment.
- Completely edentulous upper or lower jaw.
- Placement of ≥ 4 titanium implants in the relevant jaw.
- Rehabilitation with a screw-retained, one-piece full-arch fixed dental prosthesis.
- Minimum follow-up period of 6 months after prosthesis delivery.
- Regular maintenance visits at least once per year.
- Prosthetic treatment and follow-up performed by the same clinician.

### Exclusion criteria

The following exclusion criteria were applied:

- Incomplete documentation.
- Failure to comply with annual follow-up intervals.
- External surgical or prosthetic treatment.
- IFCDs with residual dentition.
- Segmented IFCDs.

### Influencing factors and variables

The following influencing factors and variables were systematically recorded and included in the statistical analysis:

- Sex: male versus female.
- Age at prosthesis delivery (years).
- History of maxillofacial tumor disease: yes versus no.
- Treated jaw: maxilla versus mandible.
- Opposing dentition: natural dentition (including tooth-supported fixed dental prostheses), mixed tooth–implant-supported fixed dentition, IFCDs, IODs, tooth–mucosa-supported removable dentures, and mucosa-supported complete dentures.
- FP-Type (Fixed Prosthesis) according to Misch et al. [15, 16]
- Presence of a cantilever.
- Veneering material: resin versus ceramic.
- Framework material: titanium versus base metal alloy versus high noble metal alloy (gold).
- Implant manufacturer/system and implant type.
- Implant connection: butt-joint versus conical connection.
- Prosthetic level: abutment level versus implant level.
- Date of implant placement.
- Date of complication (technical or biological).
- Complication region: anterior (FDI 13–23) versus posterior.
- Number of implants per IFCD.

### Data sources/measurement

Data collection was carried out between January 1, 2024, and May 1, 2024. The data sources included the electronic patient records (VISIdent^®^; BDV Dental GmbH & Co. KG) and digital radiographs from Sidexis XG^®^ (Dentsply Sirona Deutschland GmbH). Access to the data was restricted to authorized personnel only. Subsequently, all available radiographs were visually reviewed to confirm final eligibility for inclusion. No standardized quantitative radiographic measurements, such as marginal bone level changes, were systematically performed.

If a failed IFCD was replaced by a newly fabricated restoration during the study period (*n* = 4), the patient retained the original identifier to account for intra-subject correlation, which was modeled using a frailty term. These cases were analyzed as recurrent events using an Andersen–Gill counting process approach (start, stop, status), while the replacement prosthesis was treated as a new prosthetic observation unit in the prosthesis-level analyses. Follow-up for the failed IFCD ended at the time of removal and was recorded as a failure event. The patient re-entered the risk set only after delivery of the replacement IFCD. To preserve continuity of the underlying baseline hazard, entry and exit times were defined according to the actual chronological time since initial study inclusion rather than by resetting the time-to-event to zero.

### Definition of complications

Complete failure: Complications that required permanent removal of the IFCD due to severe framework fracture or loss of multiple supporting implants.

Biological complications:

- Implant loss was defined as the implant not remaining in situ throughout the entire observation period.
- Peri-implantitis: Clinically documented inflammation with progressive bone loss, requiring therapeutic intervention. Due to the retrospective nature of the present study, the classification and definition of peri-implantitis cases included in the analysis relied on peri-implantitis diagnoses that had been documented and were clinically diagnosed. In accordance with the definitions generally accepted during the 20-year inclusion period, implants were considered affected, if they exhibited an inflammatory disease of the surrounding tissues associated with progressive bone loss compared with the previous examination, increased probing depths, and peri-implant bleeding on probing and/or suppuration. All patients were monitored throughout the follow-up period by the same dentist. If any signs of inflammation, the patients were referred to the department responsible for the treatment of peri-implantitis. Only patients in which peri-implantitis treatment was deemed necessary were included in the evaluation of this retrospective study. Due to the heterogeneous and incomplete documentation of probing depths, bleeding on probing, and suppuration at follow-up appointments over the 20-year period, no additional diagnostic parameters were applied for the classification of peri-implantitis.

Technical complications were defined as any documented technical complication that required intervention but did not necessitate remanufacturing of the IFCD.

- *Veneer fracture*: Repair beyond polishing required.
- *Screw loosening*: Any diagnosed loss of preload, requiring abutment screw retightening.
- *Screw fracture*: Fracture of an abutment- or framework-screw requiring clinical or laboratory intervention.
- *Framework fracture*: Fracture of the prosthetic framework of the IFCD.

### Statistical methods

Data were analyzed descriptively using SPSS (Version 29, IBM Corp.), and the subsequent analyses were performed using R (Version 4.2.3, survival package).

Descriptive statistics were used to summarize the dataset: quantitative variables are reported as mean, standard deviation, and minimum/maximum values, whereas qualitative variables are presented as absolute and relative frequencies. Group comparisons were conducted using independent-samples *t*-tests when normal distribution was confirmed by the Kolmogorov–Smirnov test. For survival analysis, Kaplan–Meier curves were generated to estimate survival rates, and groups were compared using the log-rank test. Within the multivariable analysis, the association between risk factors (e.g., veneering material) and time to recurrent prosthetic or implant complications was assessed using Cox proportional hazards regression for recurrent events according to the Andersen–Gill model, complemented by a frailty component to account for intra-individual correlations. Mean cumulative function (MCF) curves were used to descriptively illustrate the cumulative burden of recurrent prosthetic and implant-related complications over time. In total, 251 risk periods were modeled, and complication-free courses were treated as censored observations. Hazard ratios were obtained from multivariable models including age, sex, history of tumor disease, veneering and framework materials, and jaw location. Model performance was evaluated using the concordance index (C-index) and the likelihood ratio test. To ensure a precise multivariate model, potential covariates (e.g., FP-Type, cantilever length, opposing dentition, and implant system) were first screened via univariate analysis. Variables were excluded from the final Andersen-Gill Cox models if they exhibited high homogeneity and univariate screening showed no significant association with the outcome. Associations between categorical baseline characteristics and material groups were assessed using Pearson’s chi-square or Fisher’s exact tests as appropriate (depending on expected cell counts of the contingency table).

Statistical significance was set at *p* < 0.05.

## Results

### Descriptive data

**Fig. 1:**
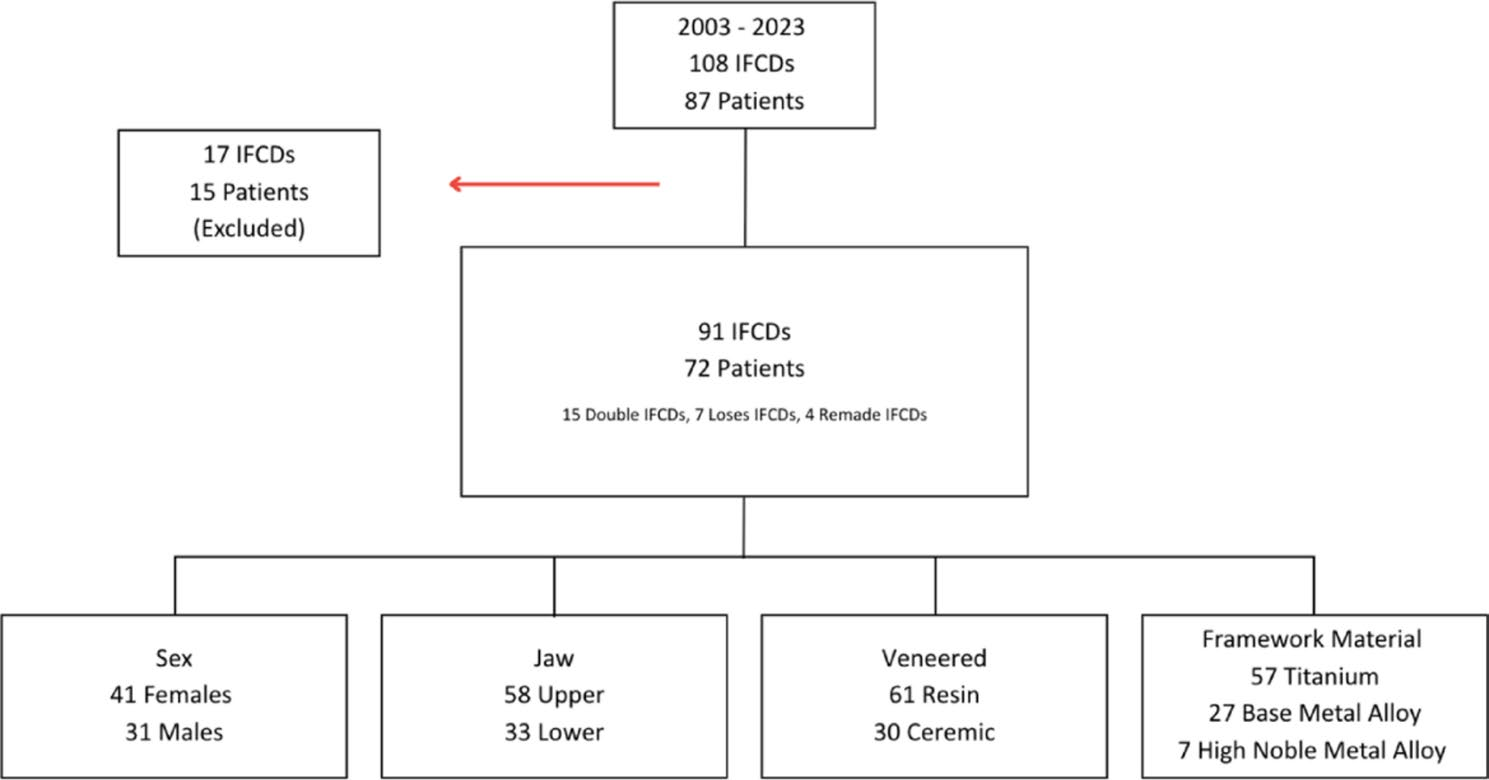
Flowchart of patient and restoration inclusion for IFCD cases (2003–2023)

Between 2003 and 2023, a total of 108 IFCDs were delivered to 87 patients. Of these, 17 IFCDs in 15 patients were excluded due to incomplete documentation or failure to meet the inclusion criteria. During the osseointegration period, eight implants were lost and subsequently explanted prior to prosthetic delivery, and were therefore excluded from further analysis. Consequently, 91 IFCDs in 72 completely edentulous patients and 498 implants were included in the final analysis (Fig. 1).

The study population consisted of 41 females (56.9%) and 31 males (43.1%). At the time of prosthesis delivery, the mean patient age was 63.1 years (SD 11.4; range 26–86 years). The mean follow-up period was 6.8 years (SD 5.0; range 0.5–17.3 years). Straumann (Straumann AG, Basel, Switzerland) were the most frequently used implants, with twelve IFCDs supported by Straumann Standard implants, eleven by Straumann Bone Level implants, six by Straumann Bone Level Tapered implants, and four by Straumann BLX implants. This was followed by Nobel Biocare implants (Nobel Biocare Services AG, Kloten, Switzerland), with 18 IFCDs supported by NobelReplace implants and 14 by NobelActive implants. Fourteen IFCDs were supported by blueSKY implants (bredent medical GmbH & Co. KG, Senden, Germany). In total, ten IFCDs were restored on CONELOG implants (ALTATEC GmbH, Wimsheim, Germany), including six Screw-Line and four Progressive-Line implants. One IFCD each was supported by Xive implants (Dentsply Implants Manufacturing GmbH, Mannheim, Germany) and ICX implants (medentis medical GmbH, Bad Neuenahr-Ahrweiler, Germany). In the entire cohort, 40 IFCDs were followed up for less than 5 years, while 28 IFCDs reached an observation period of 10 years or more. In total, 58 maxillary IFCDs (63.7%) and 33 mandibular IFCDs (36.3%) were delivered. Among the 72 patients, 15 received restorations in both jaws and four IFCDs were replacements after failure. It was known that at the time of data analysis, six patients had died, with their IFCDs remaining in situ. Thirteen patients with medical history of cancer (a total of 15 IFCDs) were included. Complete data were available for all defined demographic variables across all included cases with no missing values. The number of implants per IFCD are presented in Table 1.

**Table 1:** Overview of documented implants per IFCD

Implants per IFCD	*n*	Proportion %
4 implants	33	36.3
5 Implants	7	7.7
6 Implants	42	46.2
7 Implants	1	1.1
8 Implants	5	5.5
9 Implants	1	1.1
11 Implants	1	1.1
12 Implants	1	1.1

### Technical and biological complications

During the observation period, a total of 169 complications were documented across 133 follow-up visits; multiple events per visit were possible (Table 2). Of the 91 IFCDs included, 45 remained free of complications, while 46 experienced at least one complication during follow-up. In seven patients, complete failure of an IFCD was observed; in four of these cases, a new restoration was fabricated and re-included in the analysis as a new prosthetic observation unit. An overview of all documented technical and biological complications is provided in Table 2.

**Table 2:** Overview of documented complications

Complication type	*n*	Proportion %
**Technical complications**	**118**	**69.8**
Veneering fractures	104	61.5
Screw loosening	5	3.0
Framework fractures	5	3.0
Screw fractures	4	2.4
**Biological complications**	**51**	**30.2**
Peri-implantitis	30	17.8
Explantations	21	12.4
**Loss of IFCD**	**7**	**–**
**Re-made (within period)**	**4**	**–**

**Fig. 2:**
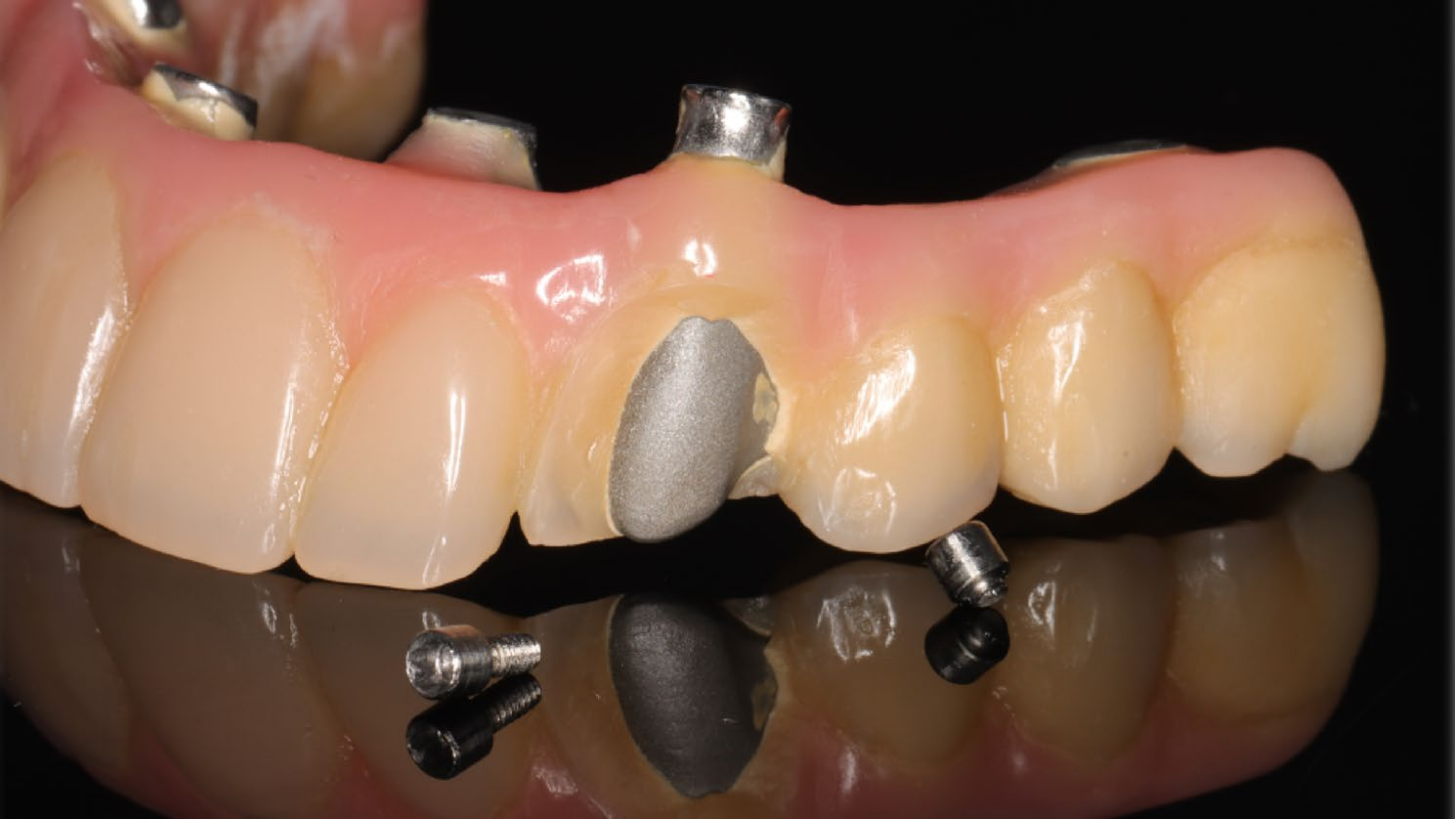
Maxillary full-arch prosthesis (titanium–resin) showing veneering and screw fractures

Technical complications predominated, with veneer-related events representing the most frequently observed technical issue. Veneer fractures affected 32 IFCDs, whereas 59 IFCDs remained free of any veneer-related complications throughout the entire observation period (Fig. 2).

With regard to biological complications, peri-implantitis was observed in 14 IFCDs, while the remaining 77 IFCDs did not require any peri-implantitis–related therapeutic intervention during follow-up. Overall, over a mean observation period of 6.8 ± 5.0 years (range, 0.5–17.3 years) 21 of 498 implants (4.2%) were explanted.

#### Overall prosthetic survival of IFCDs

**Fig. 3:**
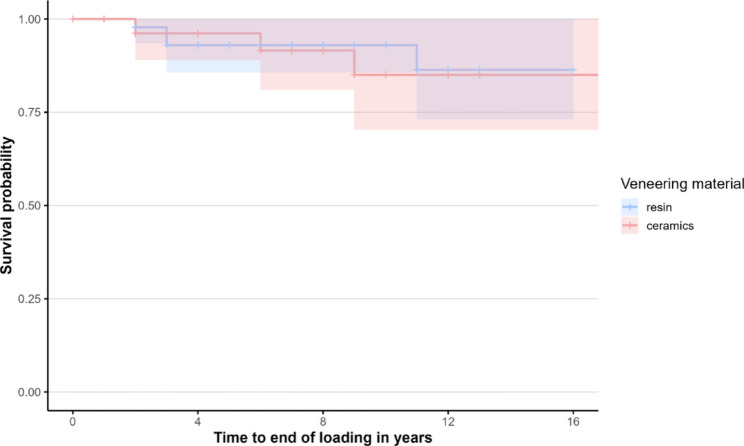
Kaplan–Meier curves for time to end of loading of IFCDs (resin vs. ceramic)

Seven IFCDs failed, corresponding to a cumulative overall IFCD survival of 92.3%. For the Kaplan–Meier analyses, the time to the first event was considered, while recurrent events were accounted for using the Andersen–Gill model. Overall survival of the IFCDs (complete failure, Fig. 3): The survival curves demonstrated comparable trends between RV and CV. The probability of survival without complete failure for RV was 93.0% (95% CI [85.7%, 100.0%]) and 85.0% after both five and ten years, and 96.2% (95% CI [89.0%, 100%]) and 85.0% (95% CI [70.3%, 100%]) for CV after five and ten years, respectively (*p* = 0.85).

#### Complications according to veneering material

The mean observation period was 6.3 ± 5.0 years (range 0.5–16.8) for resin-veneered prostheses (RV) and 7.9 ± 4.9 years (range 0.5–17.3) for ceramic-veneered prostheses (CV).

For RV, a significantly higher mean number of veneer fractures per IFCD was observed in the maxilla (2.03 ± 2.91) compared to the mandible (0.91 ± 1.83) (*p* = 0.004). Female patients exhibited a mean of 1.68 ± 2.53 veneer fractures per IFCD, whereas male patients showed 1.42 ± 2.78 fractures per IFCD (*p* = 0.586).

With regard to the region of complication, veneer fractures occurred significantly more frequently in the anterior region (1.27 ± 2.38 per IFCD) than in the posterior region (0.33 ± 0.86 per IFCD) (*p* < 0.001).

In contrast, CV-IFCDs showed markedly lower complication counts overall, with only minor differences between anatomical regions and without statistically significant variations among the analyzed subgroups (Table 3).

**Table 3:** Veneering fractures per IFCD according to jaw region, sex, and position

Category	*n* (IFCDs RV)	Fractures/IFCDs RV		*n* (IFCDs CV)	Fractures/IFCDs CV	
*Jaw region*						
Maxilla	37	2.03 (± 2.91) (min. 0, max. 11)	*p* = 0.004	21	0.24 (± 0.63) (min. 0, max 2)	*p* = 0.687
Mandible	23	0.91 (± 1.83) (min. 0, max. 8)		10	0.30 (± 0.68) (min. 2, max. 2)	
*Sex*						
Male	19	1.42 (± 2.78) (min. 0, max. 11)	*p* = 0.586	19	0.32 (± 0.67) (min. 0, max 2)	*p* = 0.256
Female	41	1.68 (± 2.53) (min. 0, max. 8)		12	0.17 (± 0.58) (min 0, max. 2)	
*Position*						
Ant./post.	60	1.27 (± 2.38) (min. 0, max. 11) 0.33 (± 0.86) (min 0, max. 3)	*p* < 0.001	31	0.16 (± 0.45) (min. 0, max. 2) 0.10 (± 0.40) (min. 0, max. 2)	*p* = 0.268

#### Veneering fracture–free survival

**Fig. 4:**
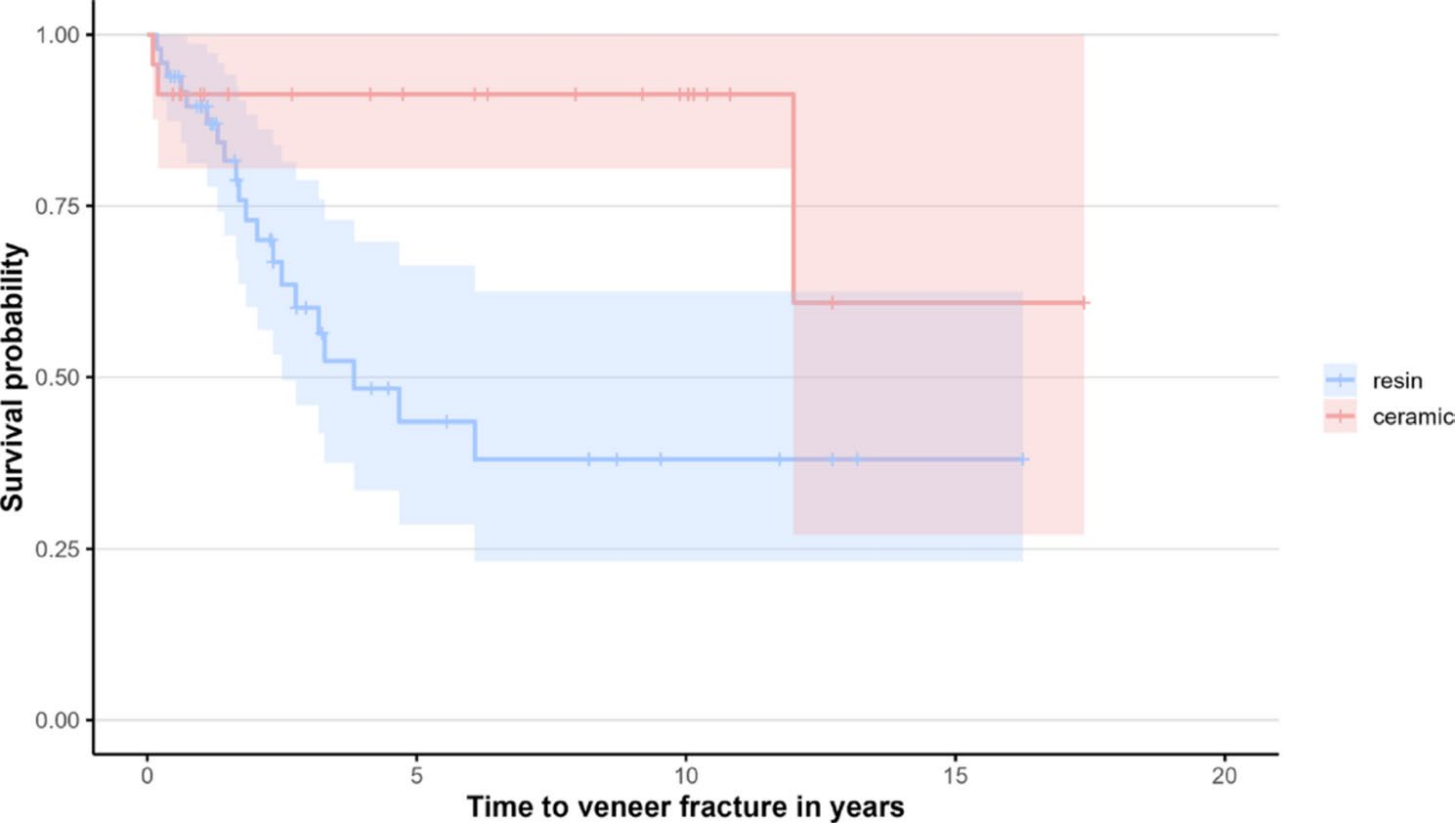
Kaplan–Meier curves for veneering fracture–free survival (resin vs. ceramic)

Significant differences were observed in favor of the ceramic group (*p* = 0.0094). The probability of remaining free from veneer fracture was 43.5% (95% CI [28.6%, 66.3%]) and 38.1% (95% CI [23.2%, 62.5%]) for RV after five and ten years, respectively, whereas for CV it remained at 91.3% (95% CI [80.5%, 100%]) for both five and ten years (Fig. 4).

#### Influencing factors (Cox regression, Andersen–Gill model with frailty)

Age at the time of prosthesis delivery showed no statistically significant association with complication risk (HR = 1.013; 95% CI 0.999–1.027; *p* = 0.065), neither sex (HR = 0.816; 95% CI 0.470–1.417; *p* = 0.469), history of tumor disease (HR = 0.786; 95% CI 0.419–1.474; *p* = 0.453), nor jaw location (HR = 0.841; 95% CI 0.501–1.412; *p* = 0.514) showed a significant influence.

#### Recurrent veneer fractures according to veneering material

**Fig. 5:**
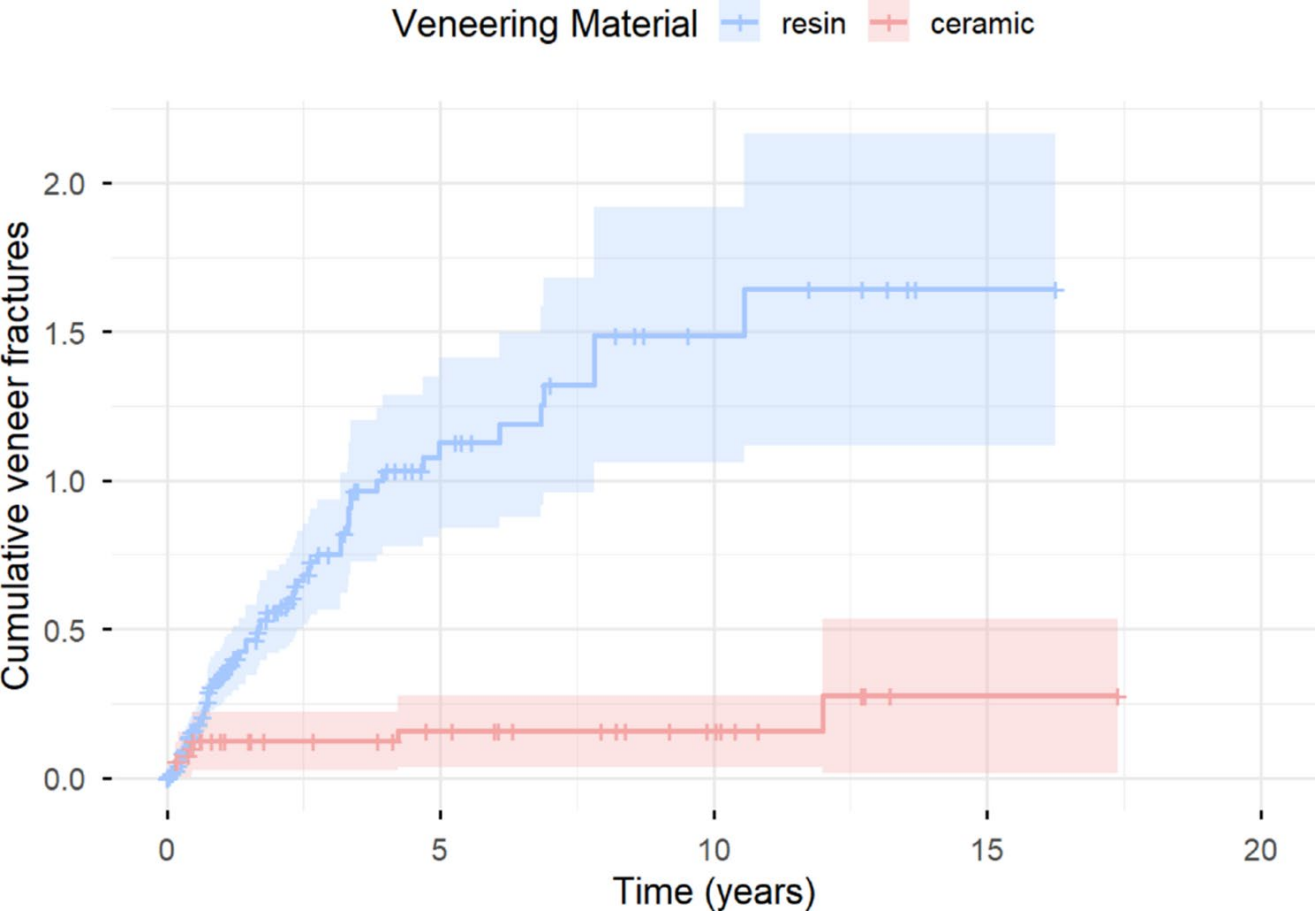
Mean cumulative function (MCF) for recurrent veneering fractures (resin vs. ceramic)

Recurrent event time analysis revealed a clear and statistically significant difference between the veneering materials (*p* < 0.0001, Fig. 5). The resin group exhibited a mean cumulative function (MCF) of 1.11 (95% CI 0.86–1.43) and 1.45 (95% CI 1.10–1.93) after five and ten years respectively. In contrast, the ceramic group showed a significantly lower MCF of 0.15 (95% CI 0.07–0.33) after both five and ten years. CV-IFCDs demonstrated a significantly higher rate of complication-free survival throughout the entire observation period compared with RV-IFCDs. After five years, the probability of complication-free survival was 66% (95% CI 0.54–0.81) for CV and 18% (95% CI 0.13–0.26) for RV; after ten years, it was 46% (95% CI 0.32–0.65) for CV and 13% (95% CI 0.08–0.21) for RV.

#### Effect of the veneering–framework material combination

The group with high noble metal alloy frameworks (*n* = 7) was excluded from subsequent statistical analyses due to the small sample size and the particular interest in frequently used material combinations; inferential conclusions apply only to the material combinations subsequently considered.

To analyze the combined influence of the variables in more detail, two multivariate Andersen-Gill models were calculated for the occurrence of all complications and for the specific risk of veneer fractures. Comparisons were made between RV–titanium (Group 1) (*n* = 53) and CV–base metal alloy (Group 2) (*n* = 22), as those material combinations represented the majority of all IFCDs.

Group 1 exhibited a more than fourfold increased risk (HR = 4.25; 95% CI 1.74–10.41; *p* = 0.0015) with good predictive accuracy (C-index = 0.819) (214 observations, 151 complications).

**Fig. 6:**
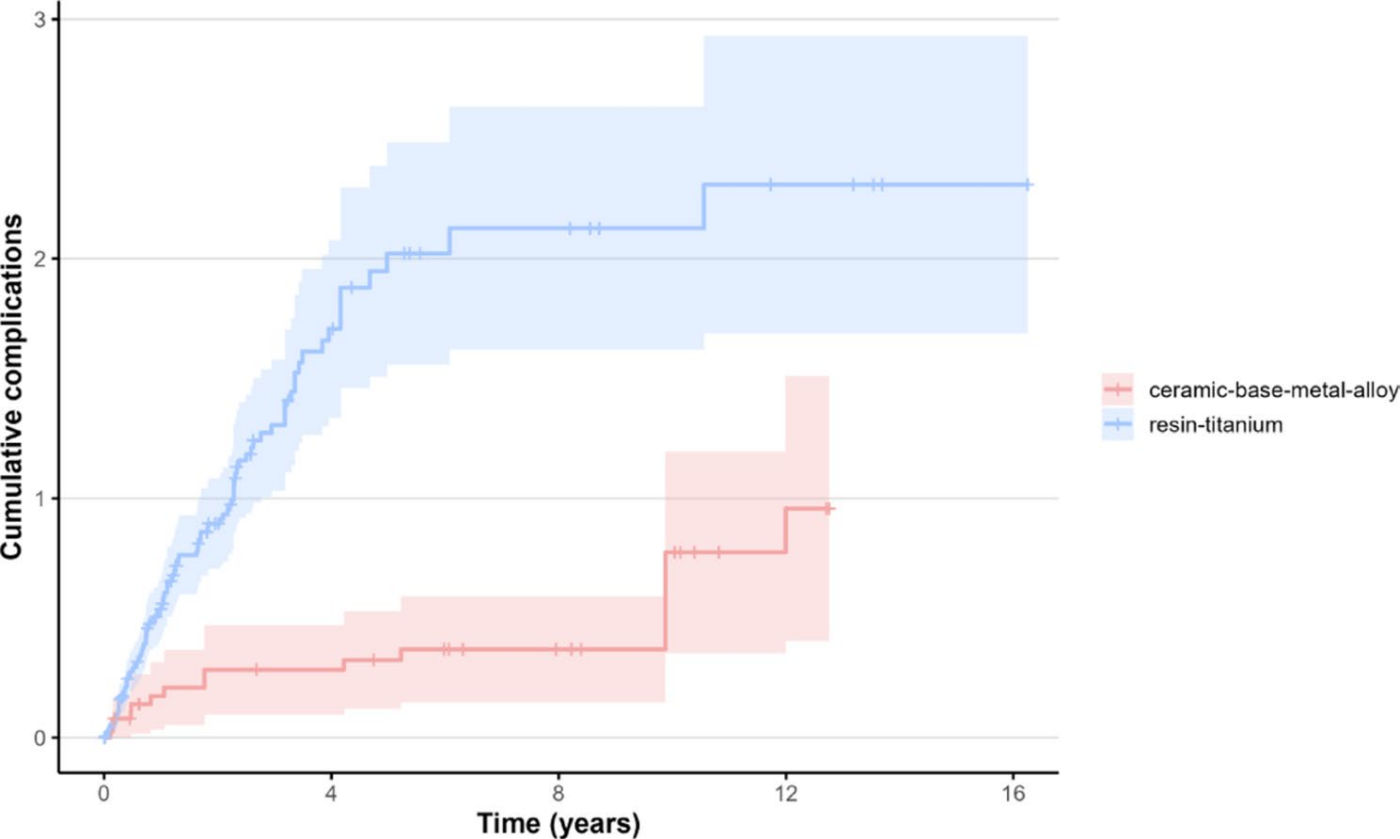
MCF for all complications in Group 1 and Group 2 restorations

The mean cumulative function for any complication for Group 1 was 1.97 (95% CI 1.57–2.48) and 2.07 (95% CI 1.63–2.63) after five and ten years respectively, while for Group 2 it was 0.32 (95% CI 0.17–0.59) and 0.69 (95% CI 0.41–1.17) respectively. After five and ten years, the survival probability for Group 2 was 85% / 85% (95% CI 0.76–0.96 / 0.76–0.96), compared with 32% / 23% (95% CI 0.24–0.43 / 0.15–0.35) for Group 1 (Fig. 6).

For veneer fractures, Group 1 showed a more than sevenfold increased risk (HR = 7.11; 95% CI 1.96–25.85; *p* = 0.0029) with very high predictive accuracy (C-index = 0.875) (214 observations, 91 veneer fractures).

**Fig. 7:**
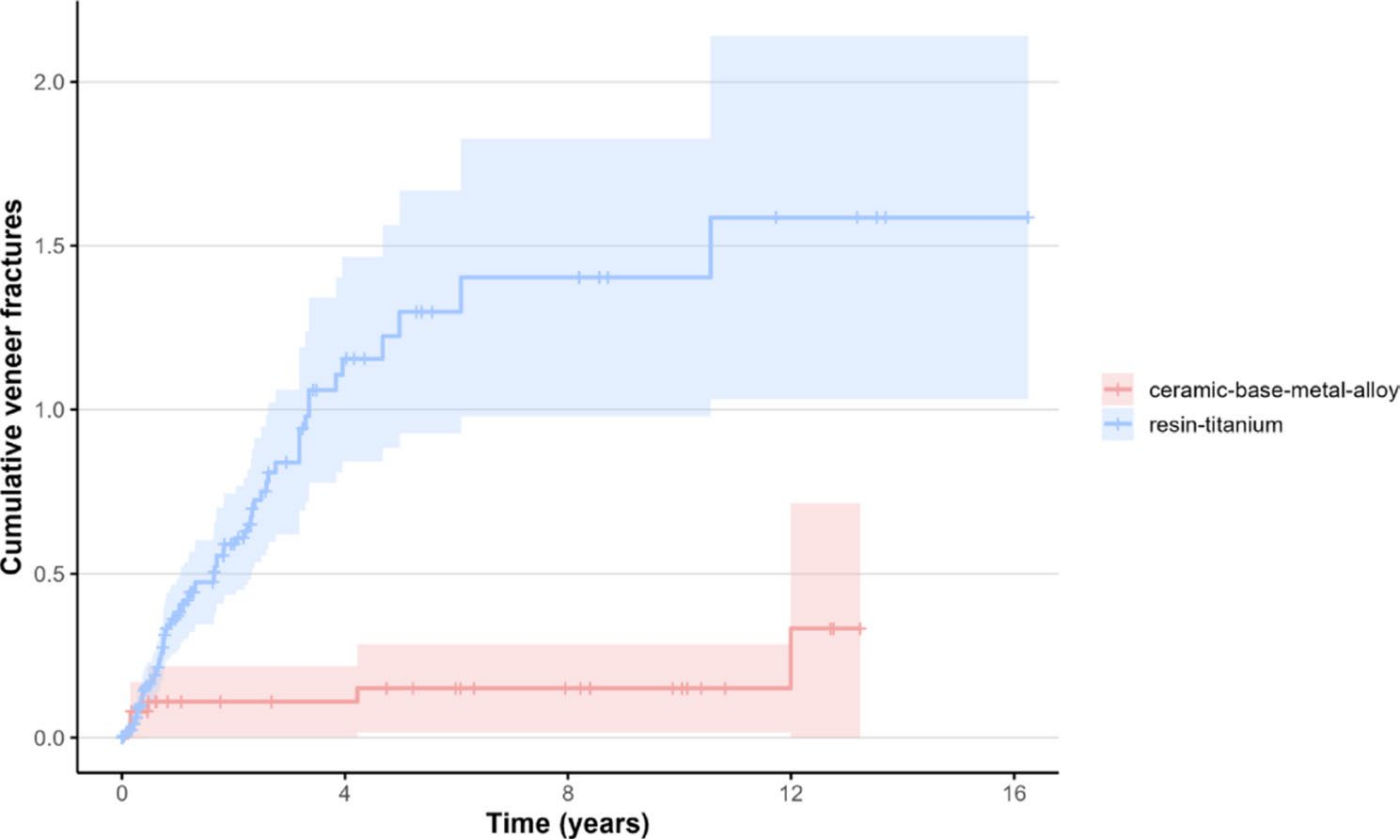
MCF for veneering fractures for Group 1 and Group 2 restorations

The mean cumulative function for veneer fractures (only) for Group 1 was 1.27 (95% CI 0.96–1.69) and 1.37 (95% CI 1.02–1.85) after five and ten years respectively, while for Group 2 it was 0.15 (95% CI 0.07–0.36) for both five and ten years (Fig. 7).

#### Further potential influencing factors

The analysis of potential influencing factors included the opposing dentition (Table 4), FP-Type, the number of implants per IFCD, the presence of a cantilever, the implant system used, the type of implant connection, and whether restoration was performed at the implant or abutment level revealed no significant effect on the risk of complications. This was true both for the overall complication risk (opposing dentition *p* = 0.985, FP-Type *p* = 0.832, implants per IFCD *p* = 0.767, cantilever *p* = 0.371, implant system *p* = 0.436, implant connection *p* = 0.654, prosthetic level *p* = 0.682) and for the material-specific risk of veneering fractures (resin veneer: opposing dentition *p* = 0.911, FP-Type *p* = 0.889, implants per IFCD *p* = 0.601, cantilever *p* = 0.063, implant system *p* = 0.183, implant connection *p* = 0.197, prosthetic level *p* = 0.197; ceramic veneer: opposing dentition *p* = 0.401, FP-Type *p* = 1.000, implants per IFCD *p* = 1.000, cantilever *p* = 1.000, implant system *p* = 0.898, implant connection *p* = 1.000, prosthetic level *p* = 0.736).

**Table 4:** Opposing dentition of the IFCDs separated into patients with and without complications

Opposing dentition	Complication-free patients	Patients with complications
Natural dentition	10	11
Mixed tooth-implant-supported dentition	15	12
IFCDs	13	14
Implant-retained removable dentures	3	4
Tooth-mucosa-supported removable dentures	2	2
Mucosa-supported complete dentures	2	3

## Discussion

This retrospective analysis over a 20-year inclusion period (2003–2023) demonstrates that IFCDs exhibit high long-term survival rates but show marked differences in complication patterns depending on veneering and framework materials. The aim of the study was to analyze the frequency of complications and survival rates of IFCDs, and to test the influence of veneering and framework materials on prosthesis survival. CVs showed a significantly longer complication-free survival compared with RVs (*p* < 0.0001), while the overall prosthesis survival did not differ (*p* = 0.85). The null hypothesis that RVs and CVs would exhibit comparable complication rates was rejected, while the null hypothesis that IFCD survival would not differ between the two groups could not be rejected. For RVs, a higher incidence of veneer fractures was observed in the maxilla compared with the mandible, as well as in the anterior region compared with the posterior region. In contrast, no significant influencing factors for the occurrence of veneer fractures could be identified for CVs.

Comparable survival rates have been reported in the literature. Papaspyridakos et al. reported five-year implant and prosthesis survival rates of 99.2% and 92.1%, respectively, for their entire cohort of IFCDs, which included metal-resin and metal-ceramic restorations [17]. Other long-term studies have likewise reported implant survival rates exceeding 96% after ten years, confirming the findings of the present study [18]. However, mechanical complications, particularly veneer fractures, have been reported to be the most common technical issues [11, 19, 20]. In the present study, veneer fractures represented the most frequent technical complication, followed by screw loosening and framework fractures. In line with our findings, CVs are commonly considered mechanically more resistant due to their greater hardness and wear resistance [21, 22]. Papaspyridakos et al. reported a five-year incidence of 49% for material wear and 8% for fractures, with metal–resin (RV) IFCDs being significantly more frequently affected than metal–ceramic (CV) restorations [18]. In another study, major acrylic fractures (61%) represented the most common reason for prosthesis replacement, whereas implant losses occurred in approximately 7% of cases [23].

The high frequency of veneer fractures in resin-veneered IFCDs is also clinically relevant from a patient-centered and economic perspective. While patient satisfaction and long-term treatment costs were not systematically recorded in the present retrospective analysis, repeated technical complications increase maintenance burden, repair frequency, and associated aftercare costs. Such events may also affect patient satisfaction, especially when esthetics, function, or the need for repeated clinical visits are involved. Accordingly, the potential advantages of resin-veneered restorations in terms of repairability should be weighed against their higher complication frequency over time. Despite the higher fracture resistance of CVs, RVs may offer functional advantages due to their elastic, shock-absorbing properties, which should help cushion occlusal forces and reduce stress at the implant–bone interface [22, 24]. They may also be more cost-effective and easier to repair, although this advantage may be offset by the need for more frequent maintenance and repairs [11, 22].

The increase in complication frequency with longer periods of function is in line with previous observations from other long-term studies [25, 26] and highlights the importance of a consistent follow-up and maintenance protocol.

Lower survival rates have been reported in the literature following radiotherapy, which are generally attributed to xerostomia, mucositis or altered mucosal conditions [27–29].

The present study could not confirm this tendency due to the small sample size and the lack of systematic data on radiation dosage.

With regard to influencing factors, studies suggest that IFCDs in the maxilla tend to exhibit lower survival rates than those in the mandible, which may be attributed to the reduced bone quality and quantity in the upper jaw [30]. Similarly, fixed antagonists (tooth- or implant-supported) generate higher occlusal loads, thereby increasing the risk of technical complications [11, 23]. According to previous studies the complication rate could increase further when both jaws are restored with fixed prostheses, which has been associated with reduced tactile sensibility of both arches [31]. In the present study, no association was observed between complication risk and the type of opposing dentition. Both the complication and complication-free groups were comparable in size, and the distribution of IFCDs and fully natural opposing dentitions was similar in both groups. Similarly, this was the case for the factors FP-Type, the number of implants per IFCD, the presence of a cantilever, the implant system used, the type of implant connection, and whether the restoration was performed at the implant or abutment level.

Overall, for clinical practice, the results suggest that treatment planning for IFCDs should not focus on prosthesis survival alone, but also on the expected maintenance burden. Although both resin- and ceramic-veneered IFCDs showed high overall survival, resin-veneered restorations, particularly titanium-resin IFCDs, were associated with a substantially higher risk of recurrent technical complications and veneer fractures. For general practitioners, this means that patients should be informed at an early stage that long-term survival does not necessarily imply a complication-free course and that material selection may strongly influence the need for repair visits and aftercare. In patients in whom reduced technical maintenance is a priority, ceramic-veneered restorations may therefore represent the more favorable option, whereas resin-veneered restorations require careful recall planning and clear communication regarding the increased likelihood of technical intervention over time. In addition, the higher frequency of veneer fractures in the maxilla and anterior region suggests that these areas deserve particular attention during prosthetic planning and long-term follow-up. From a health-economic perspective, future prospective studies should further evaluate the trade-off between higher initial investment costs and lower repair frequency over time.

One limitation of this study is the definition of peri-implantitis used. Documentation of probing depths, bleeding on probing, suppuration, and bone loss over the 20-year follow-up observation period was heterogeneous and incomplete. For this reason, patients were included in the analysis of peri-implantitis incidence not based on clear measurement data, but solely on whether peri-implantitis treatment had been performed. This may explain differences compared to similar studies. With an incidence of approximately 18%, however, the present investigation aligns with the results of comparable studies. In the systematic review and meta-analysis by Estrin et al. on the clinical outcomes of metal-ceramic and metal resin IFCDs, peri-implantitis incidence ranged from 7.14% to 36.45%, depending on whether the analysis was performed at the implant or patient level and depending on the materials used [32]. Another limitation is the heterogeneous follow-up period. With an average follow-up of 6.8 years, 28 IFCDs achieved a recall duration of more than 10 years, but there were also 40 bridges with a recall duration of less than 5 years.

A potential selection bias cannot be excluded, as all patients were treated by a single experienced prosthodontist. While direct comparative evidence for complex full-arch prosthetic rehabilitation is limited [33]. Available data suggest that operator experience may affect complication rates and long-term outcomes [34–36]. Accordingly, the favorable results observed in this cohort may partly reflect the high level of operator experience and treatment standardization and should be interpreted with caution when transferred to less specialized clinical settings.

Further limitations include the retrospective nature of data collection and the lack of systematic information on occlusion or functional risk factors (e.g. bruxism, smoking, diabetes, osteoporosis), which may potentially influence the occurrence of complications [37, 38]. Moreover, changes in treatment protocols, diagnostic pathways, materials, and techniques over the 20-year study period cannot be fully excluded and may have influenced the comparability between earlier and later cases.

The major strength of this study is the long observation period of up to 17 years in a clinical cohort of 91 IFCDs. This makes the investigation one of the few long-term studies to depict clinical outcomes of implant-supported restorations over nearly two decades. A further strength is the uniform treatment approach, as all prosthetic restorations were performed by the same experienced clinician, thereby minimizing variability in treatment procedures.

The results highlight that the material system is a key factor influencing the occurrence of complications. Strategic implant positioning with sufficient accessibility for hygiene can help reduce the risk of peri-implant diseases [39]. Further studies should systematically investigate the relationship between prosthetic components and biological complications [40].

## Conclusion

Within the limitations of this long-term retrospective cohort study, implant-supported fixed complete dentures (IFCDs) demonstrated high survival rates. However, technical complications were frequent, with veneer fractures being the predominant event, especially in resin-veneered IFCDs. Ceramic-veneered IFCDs showed significantly higher veneer fracture–free and overall complication-free survival compared with resin-veneered restorations, while overall prosthesis survival was comparable between groups. Titanium–resin IFCDs exhibited a markedly increased risk of recurrent complications. These findings indicate that veneering and framework material selection substantially influences long-term maintenance requirements and should be carefully considered during treatment planning.

## Data Availability

The research data are not publicly available due to institutional data protection regulations but may be accessed upon reasonable request from the corresponding author.
